# Quantification of Vancomycin and Clindamycin in Synovial Tissue and Bone Using Ultra-High-Performance Liquid Chromatography-Tandem Mass Spectrometry

**DOI:** 10.1097/FTD.0000000000001381

**Published:** 2025-09-05

**Authors:** Amanda J. Holst, Soma Bahmany, Angela C. J. van Dorp, Jakob van Oldenrijk, P. Koen Bos, Ewout S. Veltman, Brenda C. M. de Winter, Birgit C. P. Koch

**Affiliations:** * Department of Hospital Pharmacy, Erasmus MC, University Medical Center Rotterdam, Rotterdam, the Netherlands;; † Department of Orthopaedics, Surgery, and Sports Medicine, Erasmus MC, Univeristy Medical Center Rotterdam, Rotterdam, the Netherlands; and; ‡ CATOR, Center for Antimicrobial Treatment Optimization Rotterdam, Rotterdam, the Netherlands.

**Keywords:** clindamycin, vancomycin, periprosthetic joint infection, synovial tissue and bone, ultra-performance liquid chromatography-tandem mass spectrometry

## Abstract

**Background:**

Approximately 1–2% of patients undergoing hip or knee arthroplasty encounter a periprosthetic joint infection (PJI). Currently, the treatment involves revision surgeries and long-term antibiotic therapy. However, too low antibiotic concentrations can lead to treatment failure, whereas excessively high concentrations can lead to adverse events. Although optimal dosing is very important, there remains an insufficient understanding of the relationship between the concentrations of the plasma and antibiotics at the target site. This study aimed to develop and validate a novel ultra-high-performance liquid chromatography-tandem mass spectrometry (UHPLC-MS/MS) technique for quantifying two frequently prescribed antibiotics, vancomycin and clindamycin, in synovial tissue and bone samples.

**Methods:**

Method validation was performed based on the guidelines of the European Medicines Agency (EMA) and US Food and Drug Administration (FDA).

**Results:**

The method was found linear for vancomycin from 0.8 to 25 μg/g and for clindamycin from 0.5 to 25 μg/g in the bone and from 1 to 25 μg/g for both compounds in synovial tissue (*r*^2^ > 0.990). The results of the validation of precision, accuracy, and carry-over were all within the acceptable ranges. All clindamycin extracts were stable in the autosampler for the bone and synovial tissue samples for up to 24 hours. Vancomycin extracts were stable in synovial tissue for up to 24 hours but not in bone sample extracts.

**Conclusions:**

A method for quantifying vancomycin and clindamycin in synovial tissue and bone using UHPLC-MS/MS was developed and validated.

## Abstract 2

Supplemental Digital Content is Available in the Text.

## BACKGROUND

In the Netherlands, approximately 26,000 total knee arthroplasties and 36,000 total hip arthroplasties (THA) are implanted annually.^1^ Given the aging population and the resulting increase in the number of arthroplasty procedures, it is anticipated that this number will increase. By 2030, the number of total hip and knee arthroplasties in the Netherlands is expected to rise by approximately 98% and 59%, respectively.^2^ Approximately 1–2% of patients with total hip or total knee arthroplasties develop an infection in their prosthetic joint afterward.^3,4^ Periprosthetic joint infections (PJIs) lead to a lower quality of life and higher mortality rate.^5^

The most common treatment option for PJI is a two-stage revision procedure. During the initial surgery, the infected prosthesis is extracted, the infected tissue is debrided, and a temporary antibiotic-impregnated cement spacer is inserted whenever possible. The patients are treated with antibiotics both during and after surgery for 12 weeks. In general, during the first 2 weeks, the antibiotics are administered intravenously, and during the following weeks, they are administered orally. Approximately 6–8 weeks after the first surgery, the spacer is removed and a new prosthesis is implanted. In contrast, one-stage revision involves the removal of the infected prosthesis and immediate replacement with a new prosthesis in a single surgical procedure.^6,7^ Various studies have investigated whether there is a difference in success rates between one- and two-stage revisions and have found that infection eradication rates, patient morbidity, and mortality are similar between the 2 groups.^8,9^ Therefore, one-stage revisions are becoming increasingly popular.^7^

The effectiveness of concentration-dependent antibiotic treatments relies on achieving concentrations that surpass the Minimum Inhibitory Concentration (MIC) at the site of infection. This differs from time-dependent antibiotic treatment, where the effectiveness depends on the length of time the pathogen is exposed to the antibiotic. The therapeutic success of antibiotics is determined by their degree of penetration into the affected tissue. In PJI, the infected tissue comprises the bone, synovial tissue, and synovial fluid. The degree of penetration depends on both the patient and drug characteristics and on the targeted tissue and the severity of inflammation. Although there are many factors that influence antibiotic penetration, and therefore the effectiveness of treatment, all patients with PJI receive the same dose of antibiotic treatment. In some cases, there may be a correction for renal function, but no corrections are made based on other previously mentioned factors. Individualized treatment should be administered to increase treatment success rates.^10^

When antibiotic treatment is administered, the pharmacokinetic–pharmacodynamic relationship of the drug is often not investigated. A pharmacokinetic–pharmacodynamic (PK/PD) model considers the antibiotic concentrations in the patient and the antimicrobial effects on the pathogen and the toxicity. If the PK/PD relationship is known, serum concentrations are usually not measured to determine whether the concentration predicted by the PK/PD model matches that in the blood.^11^ When serum samples are collected, the concentrations are frequently used as surrogate indicators for the concentrations present at the site of infection, owing to challenges in directly measuring local tissue concentrations. Although these surrogate values display a degree of consistency across populations, it is important to note that actual concentrations at the infection site may significantly deviate from serum levels, potentially being higher or lower.^12,13^

Further research is required to improve our understanding of antibiotic exposure at the site of PJI after one- or two-stage revision, aiming to understand the PK/PD relationships to refine antibiotic dosage protocols. Therefore, it is essential to develop and validate a method for quantifying antibiotics at the site of infection. This method was recently developed to measure the concentrations of cefuroxime and flucloxacillin in bone and synovial tissue samples.^14^ In addition to cefuroxime and flucloxacillin, vancomycin and clindamycin are 2 commonly administered antimicrobial agents during the two-stage revision. Several methods have been reported for determining vancomycin and clindamycin levels in bone and tissue samples. To measure vancomycin concentrations in bone, methods such as fluorescence polarization immunoassays,^15–18^ high-performance liquid chromatography (HPLC),^19^ ultra-high-performance liquid chromatography (UHPLC),^20^ and liquid chromatography-tandem mass spectrometry (LC-MS/MS)^21^ have been reported. Clindamycin concentrations are measured using agar diffusion.^22–24^ Although there are several methods to measure the concentrations of vancomycin and clindamycin in bone, no method has been reported using ultra-high-performance liquid chromatography-tandem mass spectrometry (UHPLC-MS/MS). UHPLC has the same operating principle as HPLC but uses small particle size columns and operates at higher pressure, leading to increased speed, sensitivity, and resolution.^25,26^ A method to quantify antibiotic concentrations in blood and synovial fluid has previously been developed for vancomycin and clindamycin using UHPLC-MS/MS.^27^ However, as synovial tissue and bone are also infected matrices, our aim was to validate a method for quantifying vancomycin and clindamycin concentrations in synovial tissue and bone with the intention of applying this method in a clinical setting. To the best of our knowledge, this is the first study to describe an analytical method for the simultaneous quantification of vancomycin and clindamycin in synovial tissues and bones using UHPLC-MS/MS.

## MATERIALS AND METHODS

### Chemicals and Reagents

Clindamycin hydrochloride was purchased from Santa Cruz Biotechnology (Dallas, TX) and Sigma-Aldrich (Zwijndrecht, the Netherlands). Vancomycin hydrochloride was purchased from Cayman Chemical (Uden, the Netherlands) and Sigma-Aldrich (Zwijndrecht, the Netherlands). Biosolve BV (Valkenswaard, the Netherlands) was of methanol LC-MS grade. Vancomycin-D_10_, an isotope-labeled internal standard (ISTD), was procured from NucleoSyn (Olivet, France), and vancomycin-D_12_ was procured from Alsachim (Illkirch, France). A Milli-Q Advantage A10 system (Merck Millipore, Darmstadt, Germany) was used to generate deionized water (Milli-Q). Blank synovial tissue and bone samples were obtained from orthopedic surgeons during primary hip or knee arthroplasty.

### Stock and Working Solutions

Stock solutions of clindamycin (1000 mg/L), vancomycin (1000 mg/L), vancomycin-D_10_ (500 mg/L), and vancomycin-D_12_ (100 mg/L) were created to prepare spike and working solutions for the calibration standards (CSs), quality controls (QCs), and ISTD. Distinct stock solutions were prepared for the CSs, QCs, and ISTD (see **Appendix**, **Supplemental Digital Content 1**, http://links.lww.com/TDM/A887). To prepare the working solution for the ISTD of vancomycin-D_10_, 1000 μL of the stock solution was diluted in 100 mL of 0.1 M zinc sulfate, yielding a concentration of 5 mg/L. For vancomycin-D_12_, 500 µL of the stock solution was diluted in 20 mL of 0.1 M zinc sulfate, yielding a concentration of 2.5 mg/L.

### Spike Solutions for Calibration and Quality Controls

To prepare the spike solutions for CSs and QCs, stock solutions were added to a 10-mL volumetric flask and then diluted using Milli-Q water. Because the spike solutions required both clindamycin and vancomycin, the volumes of each component from the stock solutions were added accordingly (see **Appendix**, **Supplemental Digital Content 1**, http://links.lww.com/TDM/A887). The concentrations of the calibration standards used for clindamycin and vancomycin in bone and synovial tissues are shown in Table 1.

**TABLE 1.** Concentrations of the Calibration Standards in μg/g for Clindamycin and Vancomycin Used for Bone and Synovial Tissue Samples

Compound	Type	Concentrations of Calibration Standards (µg/g)								*r* ^2^
		S1	S2	S3	S4	S5	S6	S7	S8	
Clindamycin (bone)	Calibration standard	0.5	1	2.5	5	10	12.5	20	25	0.990
Vancomycin (bone)	Calibration standard	0.5	1	2.5	5	10	12.5	20	25	0.996
Clindamycin (synovial tissue)	Calibration standard	0.5	1	2.5	5	10	15	20	25	0.990
Vancomycin (synovial tissue)	Calibration standard	0.5	1	2.5	5	10	15	20	25	0.995

S, standard.

### Preparation of Calibration Standards and Quality Controls

CSs were prepared by spiking 100 mg of blank synovial or bone tissues with 50 μL of spike solutions for CSs at 8 different concentrations. The QCs used for bone samples included low (2 μg/g), medium (10 μg/g), and high (17.5 μg/g) concentrations by spiking 100 mg of bone tissues with 50 μL of the spike solutions for QCs. The QCs used for synovial tissue samples were also included low (5 μg/g), medium (10 μg/g), and high (20 μg/g) concentrations by spiking 100 mg of synovial tissue with 50 µL of the spike solutions for QCs.

### Sample Collection and Preparation

#### Sample Collection

During hip or knee surgeries, orthopedic surgeons collected bone and synovial samples if the patients consented to participate in the Antibiotic expoSure aT the infection sitE in peRiprosthetic joint infeCtionS (ASTERICS) study, as described in the Clinical Application to Patient Samples section. After collection, the synovial tissue and bone samples underwent a 30-minute wash with 0.9% NaCl in an ultrasonic bath at room temperature to remove any remaining blood. Next, the samples were air-dried for 15 minutes and then frozen at −20°C. Blank synovial tissue and bone samples were immediately cleaned, air-dried, and kept frozen at −20°C until further use.

#### Sample Pulverization

Before preparing blank and patient samples for analysis, the synovial tissue and bone samples were pulverized. This process was conducted in the orthopedic laboratory using a Mikro-Dismembrator with a range of 2800–3000 rounds per min (RPM) for 30–40 seconds. After pulverization, 100 mg of the synovial tissue or bone sample was weighed into an Eppendorf tube for further analysis.

#### Sample Preparation

Before commencing the sample preparation, 50 μL of Milli-Q water was added to the patient samples. To prepare the CSs, QCs, and patient samples for analysis, 500 μL of extraction solution (composed of 50% methanol, 50% Milli-Q water, and 1% formic acid) was added to extract clindamycin and vancomycin from the synovial tissue or bone samples. Subsequently, 500 μL of the working solution ISTD was added to each sample. The samples were vortexed for approximately 60 seconds and centrifuged for 5 minutes at 1811 g (Eppendorf centrifugation). After centrifugation, 50 μL of the supernatant was transferred to an autosampler vial along with 200 μL of eluent A, described in chromatographic conditions. After mixing, the extracts were injected into a Waters Xevo TQ-S Micro UHPLC-MS/MS system for analysis.

### Chromatographic- and Mass-Spectrometry Conditions

#### Instrumentation

The analyses were conducted using a Waters Acquity UHPLC-MS/MS system (Waters Corp., Milford, MA). The UHPLC system was linked to a Waters TQ-S micro mass spectrometer equipped with a triple quadrupole and an electrospray ionization (ESI) probe. The setup also included an acuity binary solvent manager, sample manager, and column manager. Data processing was performed using Masslynx V4.1 and Targetlynx V4.1 software (Waters Corp).

#### Chromatographic Conditions

For chromatographic separation and optimization, a Waters Acquity UHPLC HSS T3 C18 column (1.8 μm, 2.1 × 100 mm) was used at a column temperature of 45°C. The mobile phase consisted of eluents A and B. Eluent A consisted of 0.1% formic acid and 2 mM ammonium acetate in 1 L of Milli-Q water, and eluent B consisted of 0.1% formic acid and 2 mM ammonium acetate in 1 L of LC-MS-grade methanol. The gradient profile was as follows: starting with 95% eluent A and 5% eluent B for the initial 0.80 minutes, transitioning to 10% eluent A and 90% eluent B over 2 minutes, and finally returning to 95% eluent A and 5% eluent B for the remaining 2.4 minutes. The total analytical runtime per sample was 5.2 minutes. Linear gradient elution at a constant flow rate of 0.35 mL/min was employed. Injection volumes were set at 1 and 10 μL for clindamycin and vancomycin for bone samples and at 10 µL for both clindamycin as vancomycin for synovial tissue samples. The autosampler temperature was maintained at +15°C. The seal wash was performed using a water–methanol mixture (9:1), with methanol serving as the needle wash.

#### Mass-Spectrometry Conditions

The ideal mass spectrometry (MS) conditions were established for each compound during the method development process. The MS conditions were operated using electrospray ionization in the positive mode (ESI+). Each analyte was prepared in a methanol solution at a concentration of 1 mg/L and directly infused into the MS/MS system. The cone voltage and collision energy were optimized individually for each analyte. The optimal MS settings were as follows: a capillary voltage of 3.0 kV, a cone voltage of 20 V, a desolvation temperature heated at 400°C with a desolvation gas flow rate of 500 L/h, and a cone gas flow rate of 10 L/h. The optimized conditions for each analyte are listed in Table 2.

**TABLE 2.** Mass Spectrometry Conditions for Clindamycin and Vancomycin

Compound	Parent ion (m/z)	Product ion (m/z)	Cone Voltage (V)	Collision Energy (eV)	Retention Time (min)
Clindamycin	425.0	125.9	2	38	1.56
Vancomycin	725.6	144.0	14	14	1.16
Vancomycin-D_10_	730.3	144.0	18	16	1.10
Vancomycin-D_12_	731.7	144.1	32	12	1.17

#### Method Validation

The method described in this article was validated in accordance with the guidelines outlined by the European Medicines Agency (EMA) and the US Food and Drug Administration (FDA) for bioanalytical method validation, as detailed in the guidelines of 2011 and 2018, respectively.^28,29^ Validation procedures were conducted to ensure the linearity, accuracy, precision, carry-over, and stability of both antibiotics in both matrices. These values met the requirements based on the guidelines of the EMA and FDA; therefore, this method was used to measure the concentrations of vancomycin and clindamycin in synovial tissue and bone of patients with PJI.

#### Linearity

The linearity of the method was validated by constructing a calibration curve comprising 8 known concentrations in duplicates. This process included measuring a blank sample without an ISTD and a zero sample with an ISTD. After applying a weighting factor of 1/x, the response (the peak area of each CS divided by the peak area of the ISTD) was plotted against the theoretical concentrations. Linear least-squares regression was employed to analyze linearity, with the determination coefficient (r^2^) required to be at least 0.990 for each calibration curve.

#### LLOQ and ULOQ

The lower limit of quantification (LLOQ) and upper limit of quantification (ULOQ) were established. The LLOQ, which represents the lowest standard of the calibration curve, was measured 5 times on 3 separate days. The LLOQ experiments were based on QCs. Accuracy and precision were required to fall within the accepted values (80–120%). The ULOQ is defined as the highest CS. The deviation from the calibration curve was required to be less than 15%.

#### Accuracy and Precision

Intraday precision was assessed by measuring 3 different concentrations, quality control-low (QC-L), quality control-medium (QC-M), and quality control-high (QC-H), 5 times within a single run. Interday precision and accuracy were also evaluated by measuring QC-L, QC-M, and QC-H 5 times on 3 different days. For precision, the relative SD (RSD) should be <15%. The accuracy was evaluated based on the bias from the measured value compared with the nominal value, and these deviations should be lower than 15%.

#### Carry-over Effect

To assess the carry-over effect, a blank sample containing ISTD was measured immediately after the ULOQ. The carry-over effect was considered acceptable if it was <20% of the LLOQ.

#### Stability

The autosampler stability was assessed by storing the extracts of the QC samples at low, medium, and high concentrations in the autosampler at 15°C for 24 hours. Subsequently, the extracts were analyzed in duplicate and against freshly prepared CSs. The concentrations measured after 24 hours were compared with those measured initially (T = 0), with acceptable recoveries within ±15%.

#### Clinical Application to Patient samples

Samples were collected from patients diagnosed with PJIs undergoing either a two-stage or one-stage revision as part of the ongoing ASTERICS study. This observational study was approved by the Medical Ethics Committee (MEC) of Erasmus MC in Rotterdam, the Netherlands (registration number: MEC-2020-0279). This study focused on evaluating the target-site concentrations of specific antibiotics administered to determine the efficacy of PJI treatment at the target-site level.

This study enrolled patients aged 18 years and older suffering from a PJI after THA or total knee arthroplasties, scheduled for either a one-stage or two-stage revision procedure. The samples were collected at 4 different time points. During the initial surgery, where the infected prosthesis was removed, samples were obtained 15–30 minutes (T1) and 90–120 minutes (T2) after intravenous antibiotic administration. Joint puncture was conducted between 3 days and 2 weeks postsurgery (T3). Finally, after 4 weeks of oral antibiotic treatment (T4), the final samples were obtained during reimplantation or joint puncture.

Vancomycin samples were collected at time points T1 and T2 for the IV-administered antibiotic, and at time point T4 to measure the remaining concentration in the bone or synovial tissue of patients who received an antibiotic spacer during the first surgery, which was removed during the second surgery. Clindamycin samples were collected at T4, 4 weeks after oral antibiotic administration during the second surgery. The measured concentrations of vancomycin and clindamycin were assessed to determine whether they exceeded the MIC_ECOFF_ (epidemiological cutoff value) for *Staphylococcus aureus*. Vancomycin has an MIC_ECOFF_ of 2 mg/L, whereas for clindamycin, the MIC_ECOFF_ is 0.25 mg/L for *S*. *aureus*.^30^

## RESULTS

### Method Validation

#### Linearity

The calibration ranges for clindamycin and vancomycin in synovial tissue and bone were validated from 0.5 to 25 μg/g. The determination coefficients (r^2^) for clindamycin and vancomycin were within the acceptable ranges for both matrices, as presented in Table 1. Linearity was deemed sufficient for both compounds in both matrices. Vancomycin-D_10_ was initially used as an ISTD to validate vancomycin and clindamycin in synovial tissue and bone. However, owing to supply issues, vancomycin-D_12_ was used for the remainder of the validation. We do not expect any problems when using these ISTDs because the only difference between them is the presence of 2 deuterium atoms.

#### Limits of Quantification

**FIGURE 1.**
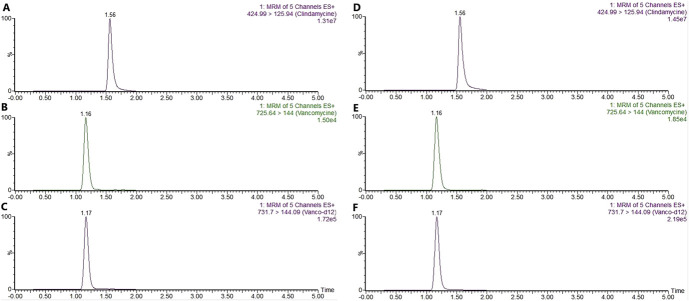
Chromatograms of the lower limit of quantification (LLOQ) samples of vancomycin, clindamycin, and ISTD vancomycin-D12 in bone and synovial tissues. A, Chromatogram of clindamycin in bone samples. The retention time in minutes is given on the top of the peak. B, Chromatogram of vancomycin in bone samples. C, Chromatogram of vancomycin-D12 in bone samples. D, Chromatogram of clindamycin in synovial tissue samples. E, Chromatogram of vancomycin in synovial tissue samples. F, Chromatogram of vancomycin-D12 in synovial tissue samples.

Different LLOQs were selected for clindamycin and vancomycin, resulting in LLOQs of 0.5 and 0.8 μg/g in bone, respectively. In synovial tissue, the LLOQ for clindamycin and vancomycin was 1 μg/g. The accuracy and precision were evaluated and found to be within the acceptance criteria, as shown in Table 3. The ULOQ was defined as the highest standard of the calibration curve (25 μg/g) for clindamycin and vancomycin in both matrices. The deviation from the calibration curve was less than 15%, which was within acceptable limits. The extracted ion chromatograms at the LLOQs are presented in Figure 1.

**TABLE 3.** Concentration, Accuracy, and Precision of LLOQ and QCs of Clindamycin and Vancomycin in Bone and Synovial Tissues

Compound	QC	Concentration (µg/g)	Accuracy (%)	Intraday Precision (%)	Interday Precision RSD (%)
Clindamycin (bone)	LLOQ	0.5	−1.2	—	11.7
	QC L	2	1.1	2.7	8.3
	QC M	10	5.6	10.3	7.7
	QC H	17.5	−2.1	4.8	12.5
Vancomycin (bone)	LLOQ	0.8	6.6	—	8.1
	QC L	2	6	6.8	6.7
	QC M	10	1.5	8.4	6.5
	QC H	17.5	6.2	3.6	5.0
Clindamycin (synovial tissue)	LLOQ	0.98	−1.6	—	9.7
	QC L	5	−1.3	5.0	6.0
	QC M	10	−7.0	7.4	8.0
	QC H	19.8	−9.9	4.5	10.9
Vancomycin (synovial tissue)	LLOQ	1	3.5	—	6.7
	QC L	5.1	−2.9	6.2	5.2
	QC M	10.2	−1.2	5.3	4.8
	QC H	20.5	−8.0	2.7	3.3

QC, quality control.

#### Accuracy and Precision

The accuracy and intra- and interday precision results are listed in Table 3. All results met the specified requirements.

#### Carry-over

The carry-over of vancomycin in the bone was determined to be 0.2%, whereas that of clindamycin was 0%. In synovial tissue, the carry-over rate for vancomycin was 0.8%, corresponding to 0.2 μg/g. The carry-over rate of clindamycin was 0%. These carry-over percentages met the specified requirements for clindamycin and vancomycin in both matrices.

#### Stability

The clindamycin extracts remained stable in the autosampler for 24 hours in the bone and synovial tissue matrix. The clindamycin extracts from the bone samples showed recoveries of 103.4% (low), 98.1% (medium), and 103.1% (high). In synovial tissue samples, the recoveries were 108.2% (low), 101.4% (medium), and 109.5% (high). Vancomycin extracts remained stable in the synovial tissue matrix, which contradicted the bone extracts. In synovial tissue, the recoveries were 88.3% (low), 103.8% (medium), and 108.6% (high). In bone extracts, the recoveries were 122% (low), 126.2% (medium), and 119.5% (high) owing to the evaporation of the extraction solvent, leading to increased extract concentrations. Therefore, vancomycin levels in bone samples should be measured directly after processing. Extracts of clindamycin-treated bone, synovial tissue, and vancomycin-treated synovial tissue samples can be analyzed up to 24 hours after processing. This is comparable to the stability results of the serum and synovial fluid extracts.^27^

#### Clinical Application to Patient samples

The concentrations of clindamycin and vancomycin were measured at the target site and in the plasma of 15 patients treated with clindamycin and 33 patients treated with vancomycin, respectively. The age range of the patients included in this study was 35–90 years old. For vancomycin, there was significant variability in concentrations between the synovial tissue and bone matrix and between patients, with particularly high concentrations measured in the synovial tissue. Specifically, 22 synovial tissue samples were collected at T1 and T2, with 6 samples falling below the validated LLOQ. Of the 22 tissue samples, 16 were above the MIC_ECOFF_ value. For the bone, 22 samples were measured at the same time points, with 4 samples below the LLOQ and 18 samples above the MIC_ECOFF_ value. Plasma samples that had been measured already were described and compared with synovial tissue and bone matrices. One synovial tissue sample showed a higher concentration than the plasma concentration. The plasma concentrations of the other samples were higher than those at the target site. In addition, samples from 22 patients were analyzed for residual vancomycin in synovial tissue and bone surrounding the vancomycin-loaded cement spacer (T4), with 13 synovial tissue samples being below the LLOQ and 1 above the ULOQ. Of the 22 tissue samples, 8 were above the MIC_ECOFF_ value 2 mg/L. In bone samples, 7 were below the LLOQ, 2 were above the ULOQ, and 12 were above the MIC_ECOFF_. Plasma analysis is still ongoing; however, for the measured samples, 2 of the 16 samples were above the LLOQ. After calculating the average concentrations of vancomycin in the plasma, synovial tissue, and bone, a reduction in the concentration in synovial tissue and bone relative to plasma was observed. The concentration in synovial tissue was reduced by a factor of 0.5 and in bone by a factor of 0.3 compared with levels in plasma. For clindamycin, out of 15 patients, 11 synovial tissue samples and 14 bone samples were below the LLOQ for clindamycin. Samples above the LLOQ were also above the MIC_ECOFF_ of 0.25 mg/L. Of the 15 patients, 3 plasma samples were below the LLOQ. The results of the clinical samples are presented in Table 4.

**TABLE 4.** The results of the Measured Clinical Samples

Compound	Patient	Plasma Concentrations (mg/L)	Synovial Tissue Concentrations (µg/g)	Synovial Tissue Concentrations (mg/L)	Bone Concentrations (µg/g)	Bone Concentrations (mg/L)
Vancomycin	1 T1	NA	< LLOQ	<LLOQ	<LLOQ	<LLOQ
	1 T2	NA	12.7	25.4	2.8	5.6
	2 T1	NA	20.6	41.2	2.3	4.6
	2 T2	NA	21.9	43.7	2.5	5
	3 T1	>ULOQ	<LLOQ	<LLOQ	1.2	2.4
	3 T2	23.1	3.9	7.8	4.6	9.2
	4 T1	45.8	1.2	2.4	3.4	6.8
	4 T2	36.7	<LLOQ	<LLOQ	3.1	6.2
	5 T1	23.1	7.6	15.2	5.9	11.8
	5 T2	18.9	1.2	2.4	1.9	3.8
	6 T1	NA	<LLOQ	<LLOQ	<LLOQ	<LLOQ
	6 T2	NA	<LLOQ	<LLOQ	<LLOQ	<LLOQ
	7 T1	25.4	5.0	10.0	11.5	23
	7 T2	17.9	1.2	2.4	6.0	12.0
	8 T1	17.0	2.5	5.0	2.1	4.2
	8 T2	15.1	1.9	3.8	2.8	5.6
	9 T1	22.5	5.0	10.0	2.5	5.0
	9 T2	22.8	12.9	25.8	7.2	14.4
	10 T1	49.2	1.2	2.4	4.2	8.4
	10 T2	20.5	12.3	24.4	4.1	8.2
	11 T1	<LLOQ	<LLOQ	<LLOQ	<LLOQ	<LLOQ
	11 T2	47.6	1.8	3.6	3.9	7.8
	12 T4	NA	5.2	10.4	<LLOQ	<LLOQ
	13 T4	NA	>ULOQ	>ULOQ	>ULOQ	>ULOQ
	14 T4	<LLOQ	<LLOQ	<LLOQ	1.8	3.6
	15 T4	NA	6.3	12.6	4.1	8.2
	16 T4	<LLOQ	2.6	5.2	14.5	29.0
	17 T4	0.61	1.0	2.0	2.5	5.0
	18 T4	<LLOQ	<LLOQ	<LLOQ	8.4	16.8
	19 T4	<LLOQ	<LLOQ	<LLOQ	2.2	4.4
	20 T4	<LLOQ	<LLOQ	<LLOQ	<LLOQ	<LLOQ
	21 T4	<LLOQ	<LLOQ	<LLOQ	<LLOQ	<LLOQ
	22 T4	<LLOQ	<LLOQ	<LLOQ	1.0	2.0
	23 T4	NA	<LLOQ	<LLOQ	5.8	11.6
	24 T4	<LLOQ	<LLOQ	<LLOQ	NA	NA
	25 T4	<LLOQ	1.5	3.0	8.8	17.6
	26 T4	<LLOQ	<LLOQ	<LLOQ	<LLOQ	<LLOQ
	27 T4	<LLOQ	1.53	3.06	<LLOQ	<LLOQ
	28 T4	<LLOQ	<LLOQ	<LLOQ	2.2	4.4
	29 T4	<LLOQ	<LLOQ	<LLOQ	1.4	2.8
	30 T4	<LLOQ	<LLOQ	<LLOQ	<LLOQ	<LLOQ
	31 T4	<LLOQ	23.3	46.6	>ULOQ	>ULOQ
	32 T4	<LLOQ	<LLOQ	<LLOQ	<LLOQ	<LLOQ
	33 T4	19.4	2.8	5.6	4.4	8.8
Clindamycin	1 T4	<LLOQ	1.2	2.4	1.3	2.6
	2 T4	<LLOQ	<LLOQ	<LLOQ	<LLOQ	<LLOQ
	3 T4	1.1	<LLOQ	<LLOQ	<LLOQ	<LLOQ
	4 T4	0.8	<LLOQ	<LLOQ	<LLOQ	<LLOQ
	5 T4	7.7	5.0	10.0	<LLOQ	<LLOQ
	6 T4	0.7	<LLOQ	<LLOQ	<LLOQ	<LLOQ
	7 T4	2.0	1.2	2.4	<LLOQ	<LLOQ
	8 T4	<LLOQ	<LLOQ	<LLOQ	<LLOQ	<LLOQ
	9 T4	2.1	<LLOQ	<LLOQ	<LLOQ	<LLOQ
	10 T4	2.2	<LLOQ	<LLOQ	<LLOQ	<LLOQ
	11 T4	2.3	<LLOQ	<LLOQ	<LLOQ	<LLOQ
	12 T4	2.5	1.2	2.4	<LLOQ	<LLOQ
	13 T4	NA	<LLOQ	<LLOQ	<LLOQ	<LLOQ
	14 T4	1.4	<LLOQ	<LLOQ	<LLOQ	<LLOQ
	15 T4	NA	<LLOQ	<LLOQ	<LLOQ	<LLOQ

T1 is time point 1, T2 is time point 2, and T3 is time point 3. T1 and T2 samples were taken during the first surgery, and T3 samples were taken during the second surgery after the patients received IV and oral antibiotics. NA indicates that the sample was not analyzed.

## DISCUSSION

A UHPLC-MS/MS method was developed and validated for measuring the concentrations of clindamycin and vancomycin in synovial tissue and bone. To the best of our knowledge, no method has been published that describe the quantification of vancomycin and clindamycin in synovial tissue and bone samples using UHPLC-MS/MS.

A limitation of this validation is that the matrix effects were not evaluated because of the complexity of the matrices involved. To assess matrix effects, multiple batches of blank materials were required. This poses a challenge, given the difficulty in obtaining blank human tissue from the target sites. Nevertheless, the CSs and QC samples were prepared from different batches of blanks during method validation, which differs from method validation that uses a single batch of blanks. Therefore, numerous synovial tissue and bone samples from various patients were examined. This ensured accuracy across different batches of tissues. Another limitation is the use of 2 different internal standards during validation. However, as mentioned earlier, because the only difference between the 2 ISTDs was the presence of 2 deuterium atoms, no problems were expected. The retention times of vancomycin-D_10_ and vancomycin-D_12_ were slightly different (Table 2). The difference in retention time between the ISTDs may be caused by changes in column performance over time.

In addition, the autosampler temperature was set to 15°C, which is the commonly used temperature level by laboratories. However, to enhance the stability testing, it can be lowered to below 10°C.

Moreover, the bone samples collected from patients typically contain both cancellous and cortical bone. Due to the different densities of these bone types, the antibiotics were not uniformly distributed within the samples. Because the samples were homogenized into a single sample, the resulting concentrations may not accurately reflect the antibiotic levels at the infection site. We recommend separating the bone samples immediately after collection into cortical and cancellous bone samples for separate analysis of antibiotic concentrations. It is important to account for both concentrations to obtain a comprehensive overview of antibiotic distribution in the bone, which will provide more precise insights in the future.^31^ It was not possible to repeat the analysis on patient samples because of the small sample size and the invasiveness of obtaining samples directly from the site of infection.

Given the number of samples falling below the LLOQ, additional analytical validation could be considered to lower the LLOQ for vancomycin. Although validation of the ULOQ is also possible, the primary clinical goal is to achieve concentrations above the MIC_ECOFFS_ of the pathogens. Another limitation is the lack of local MIC_ECOFF_ measurements, which should be addressed in future studies. Vancomycin is often used against *S*. *aureus* in cases of PJI with a MIC_ECOFF_ of 2 mg/L.^30^ Because the MIC_ECOFF_ is expressed in mg/L and the measured concentrations in tissue and bone samples are in μg/g, a conversion was applied using the spike volume (50 µL) and the tissue and bone samples weight (100 mg). This resulted in a conversion factor of 2.0, allowing the concentrations to be expressed in mg/L. Although MIC values are typically determined in plasma, the conversion of tissue and bone concentrations to the same unit (mg/L) allows for a more direct comparison. Currently, the validated concentration range for vancomycin in tissue is 1–25 μg/g, which corresponds to 2–50 mg/L. For bone, the range is 0.8–25 μg/g, corresponding to 1.6–50 mg/L. For clindamycin, given that almost all the samples were below the LLOQ, further analytical validation to lower the LLOQ is recommended. The MIC_ECOFF_ for *S*. *aureus* for clindamycin is 0.25 mg/L.^30^ The measurement range for clindamycin in tissue is also 1–25 μg/g, corresponding to 2–50 mg/L, and in bone it is 0.5–25 μg/g, corresponding to 1–50 mg/L. To measure concentrations above the MIC_ECOFF_, an additional validation of the concentration range of clindamycin in synovial tissue and bone samples is advised. Although many tissue samples were below the LLOQ for clindamycin, a significant number of samples appeared to exceed the MIC_ECOFF_ value. However, because these values fall below the LLOQ, they cannot be confirmed with certainty, highlighting the need to lower the LLOQ to obtain more definitive results. However, only a few bone samples exceeded the MIC_ECOFF_ value. This is in contrast to findings in the literature, where clindamycin is known to have good tissue penetration into the bone.^32–34^ However, most studies were conducted in the 1970s, and in most studies, patients were administered an intravenous or intramuscular injection of clindamycin instead of oral dosages.^22,24,35–38^ Further research is required to determine whether there is a difference in tissue penetration between intravenous and oral dosages.

## CONCLUSIONS

A precise method for analytical quantification of vancomycin and clindamycin in synovial tissue and bone samples using UHPLC-MS/MS was developed, optimized, and validated. The validation results confirmed that the analysis is sensitive, accurate, and precise across a concentration range of 0.5–25 μg/g for clindamycin and 0.8–25 μg/g for vancomycin in bone samples. For synovial tissue samples, the method was accurate over a range of 1–25 μg/g for both antibiotics. To the best of our knowledge, this is the first validated method for quantifying vancomycin and clindamycin in synovial tissue and bone using UHPLC-MS/MS. This method has already been applied in a clinical study, demonstrating that vancomycin and clindamycin can be measured in synovial tissue and bone samples using a UHPLC-MS/MS system. However, as many clindamycin samples fell below the LLOQ, additional validation may be considered to lower the LLOQ.

## Supplementary Material
