# Supplementary material for: Quantification of Vancomycin and Clindamycin in Synovial Tissue and Bone Using Ultra-High-Performance Liquid Chromatography-Tandem Mass Spectrometry
Source: Ther Drug Monit. 2025 Sep 5;48(3):331–9. doi: 10.1097/FTD.0000000000001381 (PMC13152068; doi:10.1097/FTD.0000000000001381)
Supplement: Supplementary file 1 [file tdm-48-331-s001.docx]

**Appendix 1:** Preparation of stock solutions for vancomycin and clindamycin.

| Component | Weighted (mg) | Volume (mL) | Solvent | Conc. (mg/L) |
| --- | --- | --- | --- | --- |
| Clindamycin | 20 | 20 | MilliQ | 1000 |
| Vancomycin | 20 | 20 | MilliQ | 1000 |
| Vancomycin-d10 | 5 | 10 | MilliQ | 500 |
| Vancomycin-d12 | 1 | 10 | MilliQ | 100 |

**Appendix 2:** Preparation of spike solutions for the calibration standards and quality controls for vancomycin and clindamycin.

| **Standard** | **From** | **Volume (µL)** | **Volume MilliQ (µL)** | **Concentration (mg/L)** |
| --- | --- | --- | --- | --- |
| 8 | Stock | 50 | 900 | 50 |
| 7 | Stock | 40 | 920 | 40 |
| 6 | Sock | 25 | 950 | 25 |
| 5 | Stock | 20 | 960 | 20 |
| 4 | S7 | 250 | 750 | 10 |
| 3 | S4 | 500 | 500 | 5 |
| 2 | S5 | 100 | 900 | 2 |
| 1.1 | Stock clindamycin | 20 | 980 | 20 |
| 1.2 | Stock vancomycin | 100 | 900 | 100 |
| 1.3 | S1.1 (clindamycin) | 100 | 9 800 | 0.2 |
|  | S1.2 (vancomycin) | 100 |  | 1 |
| QCL | Stock | 40 | 9 960 | 4 |
| QCM | Stock | 200 | 9 600 | 20 |
| QCH | Stock | 350 | 9 300 | 35 |
